# The need for general adaptive capacity—Discussing resilience with complex adaptive systems theory

**DOI:** 10.1111/risa.17676

**Published:** 2024-11-10

**Authors:** Benjamin Scharte

**Affiliations:** ^1^ International Center for Ethics in the Sciences and Humanities (IZEW) University of Tübingen Tübingen Germany

**Keywords:** adaptive capacity, complexity, resilience

## Abstract

The concept of resilience intrinsically links with both complexity and adaptive capacity. Scholars from different fields agree on this. Still, the detailed relations between resilience, complexity, and adaptive capacity need a more thorough theoretical analysis. This article analyses resilience with the help of assumptions from complex adaptive systems (CAS) theory to answer two questions in more detail: What is the relation between resilience and complexity? How can adaptive capacity contribute to resilience? By applying basic ideas from CAS theory to the resilience discourse, the article deduces that complexity of a system is a necessary condition for resilience because complex systems consist of agents that possess adaptive capacity, whereas simple systems consist of mere elements that cannot adapt to unexpected disruptions. The relation between complexity and resilience is multidimensional. Growing complexity leads to a growing need for resilience because the chances for severe, unexpected disruptions increase. The analysis of adaptive capacities revealed that systems and the agents they consist of can possess of specialized and general adaptive capacity. General adaptive capacity is the core feature of resilience because it enables systems to cope with unexpected disruptions. System design principles such as diversity within functional groups and redundancy help to increase general adaptive capacity. The same is true on the community level for social capital and on the individual level for disaster preparedness measures because they increase coping capacities independent of specific hazards.

## 1 INTRODUCTION

Research on resilience is ubiquitous (Dunn Cavelty et al., 2023). Especially in fields such as civil protection and critical infrastructure protection, resilience has become both, a research object but also a political objective. Understanding what is behind the concept and being able to depict its specifics is therefore essential. A prominent feature in resilience research is the link between resilience and complexity (Rosenow, 2012). Many scholars understand resilience as a mechanism for coping with complexity (Berkes, 2007; Brand & Jax, 2007; Chandler, 2014; Folke, 2006; Holling, 1973; Hollnagel et al., 2006; Walker, 2020; Woods, 2019). At the same time, most resilience scholars see adaptive capacity as the essential characteristic of resilience (Alexander, 2013; Edwards, 2009; Folke et al., 2002; Folke, 2006; Holling, 1973; Hollnagel et al., 2006; Luthar & Cicchetti, 2000; Provan et al., 2020; Walker et al., 2004; Walker, 2020; Woods, 2005, 2015, 2018). This article shares both assumptions. However, there is a need for a thorough theoretical analysis of the complex relations between resilience, complexity, and adaptive capacity. Although these concepts are dominant in different strands of resilience research, there is a lack of a sound theoretical substantiation of what these concepts mean and how they are linked. To close this gap, this article takes a detailed look at resilience, complexity, and adaptive capacity with the help of complex adaptive systems (CAS) theory (Holland, 1992, 2014) to help answer two questions in more detail: What is the relation between resilience and complexity? How can adaptive capacity contribute to resilience?

A prominent definition of resilience entails the notion of “bouncing back” to an original state after a disruption (Altherr et al., 2018; Ayyub, 2015; Bruneau et al., 2003; Cimellaro et al., 2010; O'Rourke, 2007; Reed et al., 2009; Sikula et al., 2015; Wildavsky, 1988). Such a “mechanical understanding” of resilience has its benefits, specifically in applied technical research, as there is always a need to specify requirements in system design and set performance criteria against which to measure resilience (Altherr et al., 2018; Leise et al., 2021). At the same time, many scholars have criticized such an understanding of resilience as insufficient (Holling, 1996; Lorenz, 2013; Walker, 2020; Walker et al., 2004). This is, because it does not “capture the complexity, permanent change, and dynamic of systems” (Lorenz, 2013, p. 8).

Following the tradition of Holling and other scholars, this article focuses on resilience thinking as prevalent in fields such as socio‐ecological resilience research and resilience engineering research from organizational studies (Berkes, 2007; Carpenter et al., 2001; Folke, 2006; Folke et al., 2002; Gunderson & Holling, 2002; Holling, 1973, 1996; Hollnagel et al., 2006; Provan et al., 2020; Walker, 2020; Walker et al., 2004; Woods, 2005, 2015, 2018, 2019; Woods et al., 2017). Their basic commonality—despite being very distinct fields—is the importance of complexity when analyzing resilience. The starting point for the following discussion will be a complexity‐informed resilience definition from socio‐ecological research:

A resilient system responds to a disturbance by changing the relative amounts of its different parts and how they interact, thereby changing the way it functions. It stays the same kind of system by learning from a disturbance, to be able to better cope with a similar disturbance in the future. It does not bounce back to look and behave exactly like it did before. Resilient systems are learning systems (Walker, 2020).

To make use of the lessons they learn, systems need adaptive capacity. For much of resilience research, adaptive capacity is central. Folke, for example, states that “resilience provides adaptive capacity” (Folke, 2006, p. 259). One of the most outspoken proponents of the centrality of adaptive capacity for resilience is Woods, who characterizes resilience as a verb “that refers to capabilities that build and sustain the potential for continuous adaptability” (Woods, 2018, p. 171). Applying central assumptions from CAS theory can help to further enrich this conceptual thinking on resilience, complexity, and adaptive capacity. To answer the two questions given above, the next section will introduce CAS theory. The article will then apply it to resilience. Regarding complexity, this will include an analysis on how complexity constitutes a need for resilience, whether increasing complexity leads to increasing resilience, and the necessity of complexity for resilience. Regarding adaptive capacity, the article will distinguish between two different forms of adaptation and discuss their prevalence and effects in complex systems.

## 2 THEORETICAL BACKGROUND—COMPLEX ADAPTIVE SYSTEMS THEORY

CAS theory was developed at the Santa Fe Institute in Santa Fe, New Mexico. Many CAS scholars come from physics or the computer sciences (Dillon, 2001; Urry, 2005). Besides formulating a theory of complex systems as such, a main goal for the Santa Fe Institute was to identify mathematically formalizable rules for modeling and simulating CAS (Urry, 2005). The language to describe the theory is formalized (Holland, 1992, 2014; Urry, 2005). The objective of this article is not to develop mathematical rules but to apply elements of CAS theory to conceptual resilience thinkingto better understand what the concept can mean in fields such as civil protection and critical infrastructure protection.

Complexity scholars distinguish between simple, complicated, and complex systems (Urry, 2005). Simple systems consist of specific elements. Their behavior can be explained using reductionist methods, by looking at these elements and aggregating their behavior. They follow deterministic, causally unambiguous rules (Grabowski & Strzalka, 2008). Complicated systems are essentially simple systems, but they consist of an unmanageable number of elements and difficult, yet still linear and deterministic, causal relationships (Grabowski & Strzalka, 2008; Urry, 2005). The central characteristic of complex systems is emergence (Mitchell, 2009; Siegenfeld & Bar‐Yam, 2020; Urry, 2005). An emergent property of a system cannot be explained by looking at its individual elements, but only on a systemic level (Holland, 2014; Siegenfeld & Bar‐Yam, 2020). CASs, “complex systems” for the remainder of this article, consist of “agents” that possess adaptive capacity, which they use to interact with other agents in the system and the environment (Ahmed et al., 2005; Holland, 2014). The distinction between complicated and complex systems is not clear‐cut in practice. Whether a system is defined as complicated or complex depends on how the system boundaries are drawn by the system designer or the observer, on the degree of detail in the observation, and on the underlying interest in knowledge.

The agents in complex systems are heterogeneous. They differ in roles, attributes, capacities, and so on. These agents should not be mistaken for humans. They do not necessarily possess consciousness. An “agent” is a way to conceptualize the elements of complex systems as different from the mostly invariable elements in simple and complicated systems. Elements in complex systems have agency, and they can change and adapt in response to internal and external stimuli. Thus, such agents can be humans but do not need to be humans. Agents follow specific rules on which they base their actions. Even if individual agents have simple behavioral rules, the combination of many autonomously deciding agents results in a complex system (Bankes, 2002; Holland, 2014; Lempert, 2002). This is due to so‐called feedback loops (Holland, 2014; Miller & Page, 2009). Feedback loops mean interaction between agents. The agents adapt their behavior over time in response to others, which in turn react to changes in the behavior of the first agent, and so on (Miller & Page, 2009). In addition, agents also respond to changes in the system environment. This interaction leads to emergence (Holland, 2014; Miller & Page, 2009; Mitchell, 2009).

Agents base their behavior on a set of specific signal processing rules (Holland, 2014). They rate these rules with respect to their usefulness (“credit‐assignment”) (Holland, 2014, p. 26). Agents assign a “strength” to the rules and distinguish strong from weak rules, where strong rules help them to achieve their goals better than weak rules (Holland, 2014). Agents can also replace weak rules with new ones (“rule‐discovery”) (Holland, 2014, p. 29). The question is how to assess the potential usefulness of new rules. For this purpose, agents can use their experience accumulated up to the relevant point in time. CAS theory introduces the notion of “building blocks” that agents can use in choosing meaningful new rules (Holland, 2014, p. 30). A building block consists of courses of action or outcomes that have already proven useful in various cases by following strong signal processing rules. Accordingly, adaptive capacity is demonstrated by combining different, known building blocks in such a way that strong signal processing rules result (Holland, 2014).

Agents in complex systems act based on bounded rationality (Holland, 2014; Lempert, 2002). Bounded rationality means that agents try to optimize their behavior in accordance with the information they have (Lempert, 2002; Miller & Page, 2009). With respect to resilience, it is important to note that there “are political, cognitive, informational, cultural and resource barriers to being able to prevent every possible threat” (Boin & McConnell, 2007, p. 52). Rather such threats can be unintended consequences of the interaction of agents that try to optimize their behavior under the restriction of limited information (Steen & Aven, 2011). The possibility to understand and control nonlinear interaction processes that determine complex system behavior is limited (Bankes, 2002; Urry, 2005).

Over time, agents in complex systems learn how other agents and the environment act or react to their own actions and adapt their actions accordingly (Cairney, 2012; Miller & Page, 2009). Following CAS theory, this constant adaptation leads to systems with very diverse agents, as it cannot be assumed that all agents adapt in the same way (Holland, 2014; Siegenfeld & Bar‐Yam, 2020). Holland argues, in line with Adam Smith, that the division of labor within a system increases its productivity because specialists are better able to perform their specific, small‐scale task than generalists. Thus, a higher system performance can be achieved through the cooperation of many specialists than through the cooperation of many generalists (Holland, 2014). Ceteris paribus, greater specialization leads to an increasing need to cooperate with other agents in the system, making the system more complex (Holland, 2014).

## 3 IMPLICATIONS

### 3.1 Resilience and complexity—Complex interactions

To analyze the relation between resilience and complexity, it makes sense to look at the relation between resilience and simple or complicated systems, first. Simple and complicated systems (re)act deterministically to external influences (Grabowski & Strzalka, 2008). If a disruption occurs, the response of such a system can be determined by whether this event exceeds the specified load‐carrying limits of the system. If the load‐carrying limits are not exceeded, the system continues to operate, and the degree of functioning depends on the robustness and elasticity of the system. If the load‐carrying limits are exceeded, the system collapses and cannot continue to exist without support from the environment. Many technical devices and parts of built infrastructure are examples of such systems. Bridges or tunnels have predefined stress limits, for example, with respect to the number of cars that can pass through a tunnel, the wind speed that a bridge can withstand, or the maximum weight it can carry. The same is true for power lines and transmission towers. The latter are designed to withstand a specific wind speed. If the wind blows faster, they fail. They also fail if a malicious actor manipulates or physically destroys them. It is possible to increase the robustness of such systems, but their load‐bearing capacity is still predefined and thus a priori limited. Mechanical engineering developed sophisticated system design ideas for such structures, including calling these devices or systems “resilient” (Altherr et al., 2018; Leise et al., 2021; Pelz & Groche, 2021).

Obviously, it is not wrong to use the resilience concept in such a way. For many scholars, resilience, however, is precisely the ability to cope with unforeseen, extreme adverse events, it is “the broader capability—how well can a system handle disruption and variations that fall outside of the base of mechanisms/models for being adaptive as defined in that system” (Woods, 2006, p. 21). Simple and complicated systems are not able to do this. Simple systems—consisting of mere elements without agency—do not possess the form of adaptive capacity necessary. The examples above have shown this. Without interference from the environment, for example, the system designer or the maintenance crew, such technical devices or parts of built infrastructure cannot handle disruptions “that fall outside of the base of mechanisms/models for being adaptive as defined in that system” (Woods, 2006, p. 21). This is not to say that technical systems as such can never show resilience. For example, it is also possible to conceptualize a bridge as a complex system, due to the physically nondeterministic interaction processes that occur between the different parts and materials that the bridge is made of. In CAS theory terms, such a bridge would constitute a complex physical system rather than a complex adaptive system (Holland, 2014). However, the examples show that following CAS theory simple and complicated systems cannot show resilience.

Complex systems, in contrast, can. In CAS terms, resilience necessitates the capability of agents to change the system by adapting their signal processing rules to new circumstances via rearranging building blocks and via interacting with other agents and creating feedback loops that take the new situation into account. Relating this back to the examples of bridges, tunnels, power lines, or transmission towers, the question of resilience becomes a question of drawing the right system boundaries. Instead of focusing on a single structure, it makes more sense to look at the traffic system or the energy system in a specific region. If individual structures fail, independent of the reason for failure, it might be possible to reroute traffic or energy. Or, if failure or changing conditions can be foreseen, maintenance crews might be able to reinforce specific structures beforehand. In such cases, the broader system—which is complex, again—has capabilities, foremost adaptive capacities, which enable it to cope with unforeseen disruptions that were not planned for or envisioned during the design process of the system. This distinguishes the broader, complex system, for example, the traffic system, from specific technical devices or individual structures, for example, a bridge. In complex systems, it is not possible to assess exact load‐carrying limits a priori. The occurrence of emergent properties and the nonlinearity of causal relationships prevent a clear definition of limits. Notwithstanding this, resilience is only possible in systems that possess the form of adaptive capacity just described. Thus, complexity is a necessary condition for resilience. The amount of complexity needed for resilience remains unclear. It is not necessarily the case that more complexity leads to more resilience.

Complex systems tend to become more complex over time because of specialization and subsequently growing need for interaction between the agents in the systems. Such highly specialized complex systems seem to be very stable, with little fluctuation of system performance (Gunderson & Holling, 2002; Holling, 1973; Siegenfeld & Bar‐Yam, 2020). This stability might be elusive. Increasing interdependencies between agents in complex systems increase the probability of systemic breakdowns (Siegenfeld & Bar‐Yam, 2020). The reason is that complex systems are prone to cascading effects (Buldyrev et al., 2010). Due to “tight couplings” in complex systems, even small changes can have drastic consequences (Perrow, 2011; Urry, 2005). This discussion on growing interdependencies in complex systems is a well‐known topic in risk and resilience studies (Eusgeld et al., 2011; Kröger, 2008; Kröger & Nan, 2014; Nan & Sansavini, 2017; Perrow, 2011). Assumptions from CAS theory help to support this discussion. Following CAS theory, highly specialized agents need each other to achieve their goals. If an agent fails to provide the other agents with what they need, these other agents might also no longer be able to uphold their functionality and so on. This results in an initially small problem quickly propagating through the tightly coupled system. The probability for such effects grows with the complexity of a system—albeit in a nonlinear fashion. The underlying characteristic is that of probability distributions for the occurrence of disruptions that have so called fat tails. They are characterized by the fact that seemingly highly unlikely extreme events happen more often than anticipated (Siegenfeld & Bar‐Yam, 2020). Such extreme events can follow as drastic, unintended consequences from small changes in the system, often only after a time lag, and they are typical examples of the highly nonlinear behavior that complex systems can exhibit due to emergence (Folke, 2006; Holland, 2014; Siegenfeld & Bar‐Yam, 2020). These characteristics of complex systems follow directly from CAS theory. Complex systems tend to become more complex over time and they are prone to fat tail behavior, making extreme, disruptive events more likely (Helbing, 2013; Park et al., 2013). Due to that, there is an increasing need for resilience.

Overall, complexity therefore is both a blessing and a curse. In a nutshell, resilience necessitates complexity necessitates resilience. Although this might sound like circular reasoning, it entails a central notion that can help to change perspective in two ways in some of the resilience discourses, for example, in civil protection and critical infrastructure protection. First, increasing complexity is often perceived solely as a challenge to cope with. Using CAS theory shows that complexity as such is a necessity for resilience and that increasing complexity might thus be useful to increase resilience. Second, the aim of many engineers active in civil protection or critical infrastructure protection is to develop “resilient” technical devices or structures. Following the theoretical reasoning of this article, it would make more sense to realize that such devices are part of bigger systems, and the aim should rather be enabling or increasing broader systemic resilience with the help of innovatively designing specific technical devices (Scharte, 2019). A prerequisite for this is that conceptual knowledge on resilience, complexity, and uncertainty is introduced more broadly into engineering education (Scharte, 2019; Winkens & Leicht‐Scholten, 2023a, 2023b).

### 3.2 Resilience and adaptive capacity—General adaptive capacity as core feature

Woods argues that adaptive capacity should not just be equated with resilience (Woods, 2006). This article follows Woods reasoning. It is not adaptive capacity per se that makes systems resilient. It is rather a specific form of adaptive capacity that is constitutive for resilience. Resilience, as defined in this article, involves successfully coping with unexpected disruptions that fall outside of previously defined load‐carrying limits of the affected system. CAS theory can help to identify the specific form of adaptive capacity needed for this.

In complex systems, agents specialize. To reach their goals, they need to cooperate. This cooperation is emergent rather than deterministic, as it is based on situational and contingent needs. In specific situations, agents respond to new, unfamiliar stimuli from outside by replacing inappropriate signal processing rules with new rules that are more likely to be useful. New rules mostly consist of a rearrangement of known building blocks that proved to be useful in the past (Holland, 2014). The more complex the system, the more different, specialized agents are part of the system. Holland even attributes predictive capabilities to agents in evolutionarily advanced complex systems (Holland, 2014). Accordingly, they can internally simulate the effects of new signal processing rules and forego real‐world implementation of inappropriate adaptation options (Holland, 2014). Based on their experiences, which have arisen in a nonlinear fashion from interactions with the other agents in the system as well as the environment, the different agents have very different building blocks that they can combine to create new signal processing rules. The more specialized agents there are, the more different building blocks exist in the system. Ceteris paribus, this increases the probability that at least some agents have suitable building blocks to respond adequately even to extremely unlikely disruptive events. By interacting with the other agents in the system, these agents can help the system to successfully adapt to the specific needs of an unexpected situation (Holland, 2014). These assumptions from CAS theory allow for supporting the claim that increasing complexity can lead to increasing resilience. An example of a very diversified system with highly specialized experts can be the health sector and, more specifically, the range of medical specialists. Scientific education and technological progress have led to a system that is—in principle—able to treat a very wide range of well‐known but also new and unfamiliar conditions, injuries, and diseases. The complex differentiation processes provide the system—but not the individual parts of the system—with many options for many different events.

But CAS theory can also help to formulate an inverted causal relation between growing complexity and resilience. Agents in complex systems adapt their signal processing rules to the requirements of their respective specialization, so that, in the long run, they will only have building blocks that are conducive to the most efficient performance of their specific task. If the system is hit by an unlikely disruptive event, most specialized agents will not have the necessary building blocks to successfully adapt to the event. This is because their adaptive capacity has also specialized. If the demands, which agents receive from other agents or from the system environment, change in an incremental and expected manner, they respond very quickly and adjust their signal processing rules accordingly. Their *specialized adaptive capacity* is ideally suited for this purpose. If the changes to which the system must respond are abrupt, severe, and unlikely, that is, if they differ from the development predicted by the agents, specialized adaptive capacity is not useful. For example, if civil protection personnel in a specific region only ever trains for one specific hazard, say a flooding event, they will be able to cope with different degrees of flooding, but not have the right capacities—cognitive, emotional, technical, and so on—to cope with a completely different hazard, say an earthquake. Thus, CAS theory can plausibly explain both, causally positive and causally negative links between complexity and resilience. The analysis even makes use of the same assumptions. The starkly different outcome results from different foci. If the focus is on the individual specialized agents and their specialized adaptive capacity, more complexity appears to lead to less resilience. For the sake of the example, one could say that highly specialized medical personnel are not able to treat conditions, injuries, or diseases outside of their field of expertise. If the focus is on diversity made possible through specialization and the multitude of different options available in the system, more complexity appears to have a positive effect on resilience. The range of diversely specialized medical personnel increases chances that there are some, who have the exact right specialization to treat the affected people.

Although specialization is the predominant strategy of actors in complex systems, CAS theory also describes so‐called generalists (Holland, 2014, p. 80). Generalists are agents that are less able to process specific resources in a utility‐maximizing way. Instead, they can process not only certain but many different resources (Holland, 2014). Such generalists have a broader range of possibly applicable building blocks that allow them to create fundamentally new signal processing rules, which suit the fundamentally new situation. Following CAS theory, such agents possess of a different form of adaptive capacity as compared to highly specialized agents. This article calls this form of adaptive capacity that is needed to cope with severe, unexpected changes *general adaptive capacity*. With respect to the example of the health sector, emergency doctors might qualify as “generalists” in the way just described. Emergency doctors learn more general skills for the acute care of completely different types of injured or sick people. They have a wider range of different, basic competencies at their disposal and are less specialized than their colleagues who focus on a specific field. Obviously, general adaptive capacity also allows for incremental improvements, albeit not as efficiently as specialized adaptive capacity. Emergency doctors can treat patients with very different needs, but in every specific case not as good as their specialized colleagues.

On the agent level, general adaptive capacity is a question of which building blocks agents can dispose of to create new signal processing rules. This depends on their way of credit‐assignment. General adaptive capacity necessitates a credit‐assignment, which values such rules as strong that lead to having many different and diverse options for action. Valuing such rules as strong might conflict with maximizing short‐term benefits, but it allows for coping with a broad range of possible changes. Bergström et al. (2009) give a good example of general adaptive capacity in an article addressing the question of how ship crews are able to deal with unknown situations. The participants of the study were divided into three groups that differed in terms of their prior experience in maritime operations. The first and second group had no or only limited practical experience. The third group consisted of mariners with several years of experience on large ships (Bergström et al., 2009). Although overall this third group performed best, it performed comparatively poorly in unexpected situations not covered by established plans and processes. The ingrained routines of this group prevented it from flexibly adapting to the situation (Bergström et al., 2009). Or in other words, their highly specialized adaptive capacity proved to be a hindrance toward resilience. The other two groups were more willing to approach the unfamiliar situations with the help of so‐called generic competencies, which they were taught between the different exercises (Bergström et al., 2009). Generic competencies are similar to general adaptive capacity. To be clear, Bergström et al. (2009) also stress the continued relevance of domain knowledge and experience in handling disruptions.

For further analysis, it makes sense to apply the distinction between specialized and general adaptive capacity to the system level. So far, it was about the different natures of agents’ adaptive capacities. At the system level, the causalities are the same. Specialized adaptive capacity of a system is helpful to maximize efficiency and smooth functioning in the uninterrupted everyday life of the system. Should this focus on specialized adaptive capacity lead to a lack of general adaptive capacity, the system might become brittle. Examples of such highly specialized but brittle systems can be local economic systems or companies that focus on single products and therefore cannot adapt to disruptive economic changes on a bigger scale (Plöger & Lang, 2016; Strambach & Klement, 2016; Wink, 2011). A well‐known case of that is Nokia and its inability to transform itself when the introduction of the smartphone changed the whole economic sector (Doz & Wilson, 2017). Overall, CAS theory reveals that to show resilience, systems need to have enough general adaptive capacity at their disposal. The adaptive capacity of a complex system is composed of the individual adaptive capacities of its agents—but in a nonlinear fashion. This means that a priori it is unclear to what amount of specialized and general adaptive capacity the aggregation of agents’ individual adaptive capacities leads. For example, from a broader systems point of view, emergency doctors are still highly specialized agents who perform a very specific function—the acute rescue and care of injured and/or ill people. In their everyday lives, emergency doctors engage in an activity that gives them precisely the skills they need to cope with a system‐threatening disruption—such as a large‐scale natural hazard. The resilience of the system thus benefits from these specialized agents. However, it is not necessarily true that ever more emergency doctors lead to ever higher resilience in the system. To avoid or minimize damage, entirely different capabilities may also be needed. Depending on the disruptive events, systems need agents with very different skills to cope successfully.

Following CAS theory, one can derive system principles, capabilities, and resources that can enable complex systems to enhance their general adaptive capacities, thereby demonstrating resilience when faced with disruptive events. The most obvious of these is diversity. Diversity is a system principle often linked to resilience (Folke, 2006; Folke et al., 2002; Holling, 1973; Walker, 2020). Diversity provides systems with options to successfully adapt to many different events. In CAS terms, diversity on the system level results from thorough specialization on the agent level, if a system consists of a great number of agents with very distinctive signal processing rules. The range of medical specialists in modern health systems is an example of such complexity‐caused diversity. Diversity and specialization appear congruent at first glance, but diversity is more than just different specializations. Diversity is also about differences in vulnerabilities toward disruptions (Folke, 2006; Walker, 2020). Complex systems consist of many different agents, and it makes sense to assume that several agents perform the same task. Such agents form functional groups (Folke, 2006; Holland, 2014). Diversity then means that functionally identical agents are different in terms of their vulnerability toward unexpected disruptions. Such diversity increases the general adaptive capacity of complex systems and thus their resilience.

However, if all agents operating in a system are highly specialized and if the system runs efficiently without spare capacity, it can become brittle. This brittleness arises because the specialized agents will not be available during a disruption as they are preoccupied with fulfilling their normal functions. Therefore, systems need agents that are freely available if a disruption necessitates rapid and unforeseen action. Moreover, these agents need to have the right signal processing rules at their disposal. Such agents are loose resources or “slack” (Goessling‐Reisemann & Thier, 2019). Depending on the respective system, such loose resources can result from different origins. One obvious example is redundancy, which is one of the most prominent system design principles in the resilience debate (Nowell et al., 2017). There are many forms of redundancy, such as backup, cross‐functionality, duplication, and cross‐check redundancy, and its effects on resilience are complex (Nowell et al., 2017). In CAS terms, redundancy can be provided through specialized agents, which makes sense for the most important, system‐critical tasks. This ensures that a substitution is in place regardless of the cause of failure. Alternatively, redundancy can be provided through generalists, which inherently increases general adaptive capacity since these agents can perform many different tasks that might become relevant during a disruptive event. For the conceptual discussion, it is important to remember that such “agents” are system elements, but not necessarily humans.

Another example is the community level, where loose resources can be provided by social capital. Research shows that social capital is decisive for community resilience (Aldrich, 2012; Aldrich & Meyer, 2015). Social capital consists of the social networks and connections that people have, for example, with friends and family, but also via their jobs, sports clubs, or their children's schools, and so on. Moreover, trust in and connections to local authorities are important aspects of social capital (Aldrich, 2012; Aldrich & Meyer, 2015). Conceptually, such social capital is an unspecific resource that can be used should the need arise—whether it is a need for transportation for immobile people during a disruptive event, a shelter, or knowing a dermatologist that can tell you Friday late afternoon that the skin rash your child brought home from day care is harmless and not smallpox. Social capital thus provides general adaptive capacity. For this, it must be available, accessible, and activatable (Schobert et al., 2023). Similarily, at the individual level, research has demonstrated that capabilities such as creativity, flexibility, improvisation skills, and the ability to think outside the box can enhance resilience. However, the relationships between the concepts are complex and ambiguous (Forgeard, 2024; Frykmer et al., 2018; Galatzer‐Levy et al., 2012; Gentili et al., 2019; Metzl & Morrell, 2008; Roux‐Dufort & Vidaillet, 2003; G. Webb, 2004; G. R. Webb & Chevreau, 2006). Such capabilities also qualify as general adaptive capacity because they work independently of the disruptive event. But also measures for individual disaster preparedness can be part of a CAS‐informed conception of resilience, for example, the stockpiling of resources (food, water, medical supplies, etc.), as this enables people to cope with many different situations, independent of specific hazards (Kohler et al., 2020; Kohn et al., 2012).

Taken together, the resilience of complex systems benefits from general adaptive capacity. Applying CAS theory to the debate on resilience‐enhancing factors demonstrates that system design principles such as diversity and redundancy, but also social capital as a general‐purpose resource and activities for individual disaster preparedness, are indeed beneficial for resilience. This is because they increase the general adaptive capacity of the affected systems.

### 3.3 Limitations of the approach

Although the analysis above provided insights for resilience scholars in different fields, it also has some limitations. For example, it does not entail normative considerations, like the question, whether it is good to be resilient or not. Research shows that there is a need for discussing this (Kaufmann, 2013). Resilience is “a highly political concept,” and it is influential in both the political and the scientific debate. Because of that, “its spread and its apparent “normalcy” need to be contested and questioned” (Dunn Cavelty et al., 2015, p. 6).

Another limitation is linked to the question whether the use of assumptions from CAS theory in this article does justice to its formalized basic ideas. CAS theory shall serve as a tool to simulate the behavior of complex systems with the help of sophisticated computer models (Holland, 1992; Miller & Page, 2009; Urry, 2005). Its assumptions and language stem from mathematics and the computer sciences (Dillon, 2001; Holland, 2014). This article does not dive deeper into the mathematical reasoning of CAS theory. Thus, there is a risk of interdisciplinary misunderstandings. This is a general concern when using complexity theory in the social sciences (Byrne, 1998; Cairney, 2012; Teisman & Klijn, 2008).

The basis for using CAS theory as analytical tool was the focus on systems of this article. Understanding systems as an umbrella term for everything from simple technical artifacts to complex socio‐technical arrangements allowed for applying CAS theory assumptions to the broader debate on resilience. Taking the systems view is in line with much of current resilience research (Dunn Cavelty et al., 2023). However, this systems view is not without criticism. By focusing on systems “it is possible to neglect social factors—most notably the variability in individuals’ and communities’ coping capacities, which drive adaptive processes and outcomes” (Dunn Cavelty et al., 2015, p. 2). The systems view could obscure attention to inherent injustices, inequalities, and more generally local diversity (Cairney, 2012; Dunn Cavelty et al., 2023).

There are more possible limitations, for example, Rosenow's general criticism on how seriously resilience scholars take the consequences of complexity (Rosenow, 2012). Nevertheless, the discussion on resilience from a CAS theory point of view provided the field with new questions that are worth working on. Moreover, there are other possibly interesting elements of CAS theory such as coevolutionary niche building, the usage of labels and tags, or the phenomenon of lever points that could help to better understand further characteristics of resilience (Holland, 2014).

## 4 CONCLUSION

This article used CAS theory to help answer two questions in more detail: What is the relation between resilience and complexity? How can adaptive capacity contribute to resilience? The answers to these questions demonstrate that the concept of resilience is inherently complex, as are its links to complexity and adaptive capacity. Using resilience as a systems concept allows for identifying underlying mechanisms and causalities that help to better understand the way complex systems work when confronted with disruptions. Central to this is the assumption that complexity is a necessary condition for resilience because only complex systems can possess of general adaptive capacity. In other words, complexity necessitates resilience necessitates complexity. Growing complexity leads to a growing need for resilience because the chances for severe, unexpected disruptions increase. To identify the core feature of resilience, this article distinguished between specialized adaptive capacity and general adaptive capacity. The general adaptive capacity of a system depends on the adaptive capacities of its agents, albeit in a nonlinear fashion. CAS theory shows how applying system design principles, such as diversity within functional groups of a system or redundancy, helps to increase general adaptive capacity. The analysis also revealed that social capital—on the community level—and measures for individual disaster preparedness—on the individual level—provide general adaptive capacity, which increases the resilience of the affected systems. Overall, the analysis showed that it might make sense to apply more assumptions from CAS theory to resilience reasoning in future work.

## CONFLICT OF INTEREST STATEMENT

The authors declare no conflicts of interest.

## FUNDING INFORMATION

This research did not receive any specific grant from funding agencies in the public, commercial, or not‐for‐profit sectors.
